# Poly[(μ_5_-3,5-dinitro­benzoato)rubidium]

**DOI:** 10.1107/S1600536811023026

**Published:** 2011-06-30

**Authors:** Yanqing Miao, Xiaoqing Zhang, Chunye Liu

**Affiliations:** a Xi’an Medical University, Department of Pharmacy, Hanguang Road No.137, Xi’an 710021, Shaanxi, People’s Republic of China

## Abstract

The asymmetric unit of the title compound, [Rb(C_7_H_3_N_2_O_6_)]_*n*_, comprises an Rb cation and a 3,5-dinitro­benzoate anion. The Rb cation is eight-coordinated by O atoms from five 3,5-dinitro­benzoate anions. On the other hand, each 3,5-dinitro­benzoate anion links five Rb cations with the carboxyl­ate groups as μ_3_-bridging. The metal atom is firstly linked by the carboxyl­ate groups into a chain along the *c*-axis direction, which is further linked by bonds between the Rb and nitro O atoms, giving a three-dimensional framework.

## Related literature

For 3,5-dinitro­benzoate complexes, see: Askarinejad *et al.* (2007 ▶); Madej *et al.* (2007 ▶). For Rb—O bond lengths, see: Cametti *et al.* (2005 ▶).
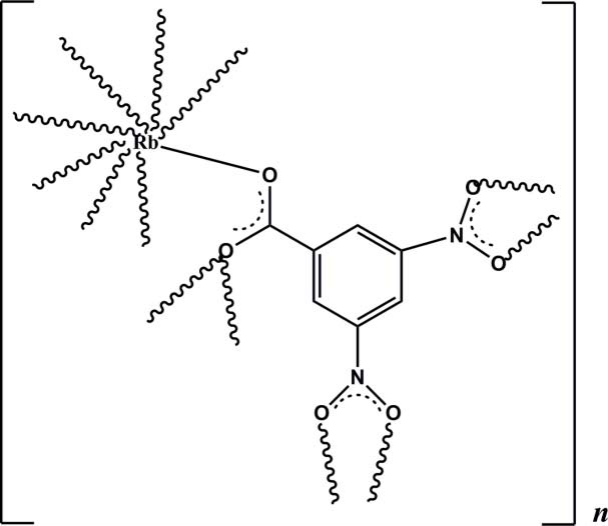


## Experimental

### 

#### Crystal data

[Rb(C_7_H_3_N_2_O_6_)]
*M* *_r_* = 296.58
Monoclinic, 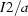

*a* = 7.2789 (15) Å
*b* = 18.072 (4) Å
*c* = 7.3652 (14) Å
β = 91.70 (3)°
*V* = 968.4 (3) Å^3^
*Z* = 4
Mo *K*α radiation
μ = 5.13 mm^−1^
*T* = 293 K
0.64 × 0.40 × 0.14 mm

#### Data collection

Bruker SMART CCD diffractometer
Absorption correction: multi-scan (*SADABS*; Sheldrick, 1996 ▶) *T*_min_ = 0.396, *T*_max_ = 1.000
4663 measured reflections
896 independent reflections
760 reflections with *I* > 2σ(*I*)
*R*_int_ = 0.053

#### Refinement

*R*[*F* ^2^ > 2σ(*F* ^2^)] = 0.033
*wR*(*F* ^2^) = 0.078
*S* = 1.06
896 reflections
75 parameters
H-atom parameters constrained
Δρ_max_ = 0.29 e Å^−3^
Δρ_min_ = −0.55 e Å^−3^

### 

Data collection: *SMART* (Bruker, 2002 ▶); cell refinement: *SAINT* (Bruker, 2002 ▶); data reduction: *SAINT*; program(s) used to solve structure: *SHELXS97* (Sheldrick, 2008 ▶); program(s) used to refine structure: *SHELXL97* (Sheldrick, 2008 ▶); molecular graphics: *SHELXTL* (Sheldrick, 2008 ▶); software used to prepare material for publication: *SHELXTL*.

## Supplementary Material

Crystal structure: contains datablock(s) I, global. DOI: 10.1107/S1600536811023026/go2012sup1.cif

Structure factors: contains datablock(s) I. DOI: 10.1107/S1600536811023026/go2012Isup2.hkl

Additional supplementary materials:  crystallographic information; 3D view; checkCIF report

## Figures and Tables

**Fig. 1.**
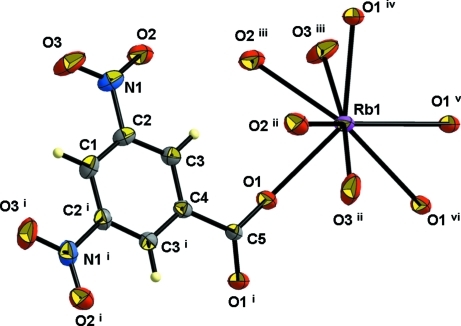
The structure of (I), showing the atomic numbering scheme. Non-H atoms are shown with 30% probability displacement ellipsoids. Symmetry codes: (i) 1.5 - x, y, 1 - z; (ii) x, 1.5 - y, -1/2 + z; (iii) 0.5 - x, 1.5 - y, 1.5 - z; (iv) 0.5 - x, y, 1 - z; (v) -1/2 + x, 1 - y, z; (vi) 1 - x, 1 - y, 1 - z.

**Fig. 2.**
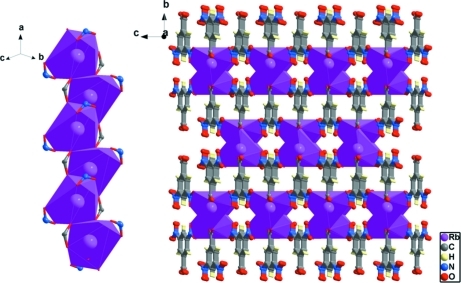
The three-dimensional framework of (I).

## supplementary crystallographic information

### Comment

In the coordination chemistry of 3,5-dinitrobenzoic acid, it has been found that the 3,5-dinitrobenzoate moiety functions as a multidentate ligand (Askarinejad *et al.*, 2007; Madej *et al.*, 2007) with versatile binding and coordination modes. In this paper, we report the crystal structure of the title compound, a new Rb complex obtained by the reaction of 3,5-dinitrobenzoic acid and RbOH in water.

The asymmetric unit of the title compound comprises a Rb cation and a 3,5-dinitrobenzoate anion. Rb cation lies on and the dinitrobenzoate is centred upon crystallographic twofold axes. The Rb cation is coordinated to eight O atoms from five 3,5-dinitrobenzoate anions (Fig. 1) with the Rb—O distances ranging from 2.761 (2) Å to 3.124 (4) Å, which are well within the range reported in the literature (Cametti *et al.*, 2005). The Rb centre is firstly linked by the carboxylic groups to give a one-dimensional chain along the *c*-axis direction, which is further linked by the phenyl groups to give a three-dimensional framework of the title compound (Fig. 2).

### Experimental

3,5-dinitrobenzoic acid and RbOH were commercially available and used without further purification. To asolution of 10 mmol 3,5-dinitrobenzoic acid in 30 ml bidistilled water, a solution of 10 mmol RbOH in 20 ml bidistilled water was added dropwise at room temperature. After vigorous stirring for 1 h, the resulting solution was then evaporated to a volume of about 15 ml in vacuum and filtered hot. The filtrate was then set aside for crystallization at room temperature. Three weeks later, colorless prism crystals of the titlecompound suitable for X-ray determination were isolated.

### Refinement

Carbon-bound H atoms were placed at calculated positions and were treated as riding on the parent C atoms with C – H = 0.93 Å, and with *U*_iso_(H) = 1.2 *U*_eq_(C).

### Crystal data

**Table:**

[Rb(C_7_H_3_N_2_O_6_)]	*F*(000) = 576
*M**_r_* = 296.58	*D*_x_ = 2.034 Mg m^−^^3^
Monoclinic, *I*2/*a*	Mo *K*α radiation, λ = 0.71073 Å
Hall symbol: -I 2ya	Cell parameters from 1610 reflections
*a* = 7.2789 (15) Å	θ = 3.0–25.4°
*b* = 18.072 (4) Å	µ = 5.13 mm^−^^1^
*c* = 7.3652 (14) Å	*T* = 293 K
β = 91.70 (3)°	Prism, colorless
*V* = 968.4 (3) Å^3^	0.64 × 0.40 × 0.14 mm
*Z* = 4	

### Data collection

**Table:**

Bruker SMART CCD diffractometer	896 independent reflections
Radiation source: fine-focus sealed tube	760 reflections with *I* > 2σ(*I*)
graphite	*R*_int_ = 0.053
φ and ω scans	θ_max_ = 25.3°, θ_min_ = 3.0°
Absorption correction: multi-scan (*SADABS*; Sheldrick, 1996)	*h* = −8→8
*T*_min_ = 0.396, *T*_max_ = 1.000	*k* = −21→20
4663 measured reflections	*l* = −8→7

### Refinement

**Table:**

Refinement on *F*^2^	Primary atom site location: structure-invariant direct methods
Least-squares matrix: full	Secondary atom site location: difference Fourier map
*R*[*F*^2^ > 2σ(*F*^2^)] = 0.033	Hydrogen site location: inferred from neighbouring sites
*wR*(*F*^2^) = 0.078	H-atom parameters constrained
*S* = 1.06	*w* = 1/[σ^2^(*F*_o_^2^) + (0.0418*P*)^2^] where *P* = (*F*_o_^2^ + 2*F*_c_^2^)/3
896 reflections	(Δ/σ)_max_ = 0.001
75 parameters	Δρ_max_ = 0.29 e Å^−^^3^
0 restraints	Δρ_min_ = −0.55 e Å^−^^3^

### Special details

**Table:**

Geometry. All e.s.d.'s (except the e.s.d. in the dihedral angle between two l.s. planes) are estimated using the full covariance matrix. The cell e.s.d.'s are taken into account individually in the estimation of e.s.d.'s in distances, angles and torsion angles; correlations between e.s.d.'s in cell parameters are only used when they are defined by crystal symmetry. An approximate (isotropic) treatment of cell e.s.d.'s is used for estimating e.s.d.'s involving l.s. planes.
Refinement. Refinement of *F*^2^ against ALL reflections. The weighted *R*-factor *wR* and goodness of fit *S* are based on *F*^2^, conventional *R*-factors *R* are based on *F*, with *F* set to zero for negative *F*^2^. The threshold expression of *F*^2^ > σ(*F*^2^) is used only for calculating *R*-factors(gt) *etc*. and is not relevant to the choice of reflections for refinement. *R*-factors based on *F*^2^ are statistically about twice as large as those based on *F*, and *R*- factors based on ALL data will be even larger.

### Fractional atomic coordinates and isotropic or equivalent isotropic displacement parameters (Å^2^)

**Table:**

	*x*	*y*	*z*	*U*_iso_*/*U*_eq_	
Rb1	0.2500	0.54657 (2)	0.5000	0.0557 (2)	
C1	0.7500	0.8732 (3)	0.5000	0.0493 (11)	
H1A	0.7500	0.9246	0.5000	0.059*	
C2	0.6036 (4)	0.83316 (17)	0.5606 (4)	0.0451 (8)	
C3	0.5991 (4)	0.75684 (17)	0.5597 (4)	0.0421 (7)	
H3A	0.4961	0.7316	0.5986	0.050*	
C4	0.7500	0.7185 (2)	0.5000	0.0387 (10)	
C5	0.7500	0.6342 (2)	0.5000	0.0398 (10)	
N1	0.4460 (5)	0.87295 (16)	0.6344 (4)	0.0595 (8)	
O1	0.6019 (3)	0.60328 (12)	0.5293 (4)	0.0649 (7)	
O2	0.3212 (4)	0.83742 (15)	0.6978 (4)	0.0696 (7)	
O3	0.4500 (5)	0.94073 (15)	0.6317 (5)	0.0913 (11)	

### Atomic displacement parameters (Å^2^)

**Table:**

	*U*^11^	*U*^22^	*U*^33^	*U*^12^	*U*^13^	*U*^23^
Rb1	0.0317 (3)	0.0382 (3)	0.0985 (4)	0.000	0.0213 (2)	0.000
C1	0.066 (3)	0.040 (2)	0.043 (3)	0.000	0.004 (2)	0.000
C2	0.0482 (19)	0.0444 (17)	0.0429 (17)	0.0112 (15)	0.0053 (14)	0.0032 (14)
C3	0.0381 (17)	0.0441 (16)	0.0441 (17)	0.0020 (15)	0.0020 (13)	0.0032 (14)
C4	0.035 (2)	0.042 (2)	0.039 (2)	0.000	−0.0038 (18)	0.000
C5	0.028 (2)	0.035 (2)	0.056 (3)	0.000	−0.0009 (19)	0.000
N1	0.068 (2)	0.0532 (18)	0.0576 (18)	0.0181 (16)	0.0117 (15)	0.0056 (15)
O1	0.0359 (13)	0.0414 (12)	0.118 (2)	−0.0037 (11)	0.0118 (13)	0.0012 (13)
O2	0.0555 (16)	0.0686 (17)	0.0860 (18)	0.0089 (13)	0.0222 (14)	−0.0045 (14)
O3	0.113 (3)	0.0515 (15)	0.112 (2)	0.0326 (16)	0.049 (2)	0.0202 (16)

### Geometric parameters (Å, °)

**Table:**

Rb1—O1	2.761 (2)	C1—C2	1.374 (4)
Rb1—O1^i^	2.925 (2)	C1—C2^iii^	1.374 (4)
Rb1—O2^ii^	3.113 (3)	C1—H1A	0.9300
Rb1—O3^ii^	3.125 (3)	C2—C3	1.380 (4)
			
O1—Rb1—O1^iv^	136.43 (9)	C1—C2—C3	122.9 (3)
O1—Rb1—O1^i^	132.92 (7)	C1—C2—N1	118.9 (3)
O1^iv^—Rb1—O1^i^	90.36 (6)	C3—C2—N1	118.2 (3)
O1^i^—Rb1—O1^v^	44.43 (9)	C2—C3—C4	118.9 (3)
O1—Rb1—O2^ii^	82.42 (8)	C2—C3—H3A	120.5
O1^iv^—Rb1—O2^ii^	68.41 (8)	C4—C3—H3A	120.5
O1^i^—Rb1—O2^ii^	120.08 (7)	C3^iii^—C4—C3	119.9 (4)
O1^v^—Rb1—O2^ii^	138.23 (8)	C3—C4—C5	120.07 (19)
O2^ii^—Rb1—O2^vi^	95.32 (11)	O1—C5—O1^iii^	126.3 (4)
O1—Rb1—O3^ii^	111.10 (10)	O1—C5—C4	116.83 (19)
O1^iv^—Rb1—O3^ii^	65.51 (9)	O1^iii^—C5—C4	116.83 (19)
O1^i^—Rb1—O3^ii^	79.74 (8)	O2—N1—O3	123.5 (3)
O1^v^—Rb1—O3^ii^	108.31 (7)	O2—N1—C2	118.9 (3)
O2^ii^—Rb1—O3^ii^	40.35 (7)	O3—N1—C2	117.6 (3)
O2^vi^—Rb1—O3^ii^	131.54 (7)	C5—O1—Rb1	164.54 (19)
O2^ii^—Rb1—O3^vi^	131.54 (8)	C5—O1—Rb1^v^	94.6 (2)
O3^ii^—Rb1—O3^vi^	171.57 (10)	Rb1—O1—Rb1^v^	89.64 (6)
C2—C1—C2^iii^	116.5 (4)	N1—O2—Rb1^ii^	93.5 (2)
C2—C1—H1A	121.8	N1—O3—Rb1^ii^	92.7 (2)
			
C3—C4—C5—O1	10.3 (2)	C3—C2—N1—O2	2.7 (5)
C3^iii^—C4—C5—O1	−169.7 (2)	C1—C2—N1—O3	2.8 (4)
C1—C2—N1—O2	−175.8 (3)	C3—C2—N1—O3	−178.8 (3)

Symmetry codes: (i) *x*−1/2, −*y*+1, *z*; (ii) −*x*+1/2, −*y*+3/2, −*z*+3/2; (iii) −*x*+3/2, *y*, −*z*+1; (iv) −*x*+1/2, *y*, −*z*+1; (v) −*x*+1, −*y*+1, −*z*+1; (vi) *x*, −*y*+3/2, *z*−1/2.

## References

[bb1] Askarinejad, A., Fadaei, M. R., Morsali, A. & Mahjoub, A. R. (2007). *J. Coord. Chem.* **60**, 753–761.

[bb2] Bruker (2002). *SMART* and *SAINT* Bruker AXS Inc, Madison, Wisconsin, USA.

[bb3] Cametti, M., Nissinen, M., Cort, A. D., Mandolini, L. & Rissanen, K. (2005). *J. Am. Chem. Soc.* **127**, 3831–3837. 10.1021/ja042807n 15771518

[bb4] Madej, A., Oleksyn, B. J. & Śliwiński, J. (2007). *Pol. J. Chem.* **81**, 1201–1218.

[bb5] Sheldrick, G. M. (1996). *SADABS* University of Göttingen, Germany.

[bb6] Sheldrick, G. M. (2008). *Acta Cryst.* A**64**, 112–122. 10.1107/S0108767307043930 18156677

